# Systemic Signs of an Unexpected Guest in a Case of Apparent Upper Gastrointestinal Bleeding Leading to an Endoscopic Extraction of a Foreign Body: A Case Report

**DOI:** 10.3390/reports8010026

**Published:** 2025-02-19

**Authors:** Rareș Crăciun, Cristian Tefas

**Affiliations:** 1 Department of Internal Medicine, “Iuliu Hațieganu” University of Medicine and Pharmacy, 8 Victor Babes Street, 400012 Cluj-Napoca, Romania; craciun.rares.calin@elearn.umfcluj.ro; 2 Department of Gastroenterology, “Prof. Dr. Octavian Fodor” Institute of Gastroenterology and Hepatology, 19-21 Croitorilor Street, 400162 Cluj-Napoca, Romania

**Keywords:** upper gastrointestinal bleeding, anaphylaxis, hymenoptera venom allergy, endoscopy, foreign body

## Abstract

**Background and Clinical Significance:** Upper gastrointestinal (GI) bleeding is a common emergency, typically requiring prompt intervention. This case report presents a unique situation where apparent GI bleeding was ultimately identified as anaphylaxis triggered by accidental wasp ingestion. Such cases are rare, underscoring the need for a broad differential diagnosis in atypical presentations. **Case Presentation:** A 53-year-old male with a history of heavy alcohol use presented with presumed acute hematemesis, hypotension, and tachycardia. An initial examination revealed mild anemia and elevated liver enzymes. An urgent upper GI endoscopy showed severe esophagitis with no signs of active or stigmata of recent bleeding; instead, two dead wasps were found in the gastric antrum. Further inquiry revealed that the patient had recently consumed a home-brewed alcoholic beverage, likely contaminated with the wasps. The patient’s symptoms were then attributed to anaphylaxis from venom exposure rather than hemorrhagic shock. The patient’s condition improved with antihistaminic therapy, and he was discharged with follow-up recommendations. **Conclusions**: This case highlights the importance of considering rare but critical diagnoses, such as insect-induced anaphylaxis, in patients presenting with presumed GI bleeding. It reinforces the value of thorough history taking, prompt endoscopy, and systematic management in assessing and treating atypical emergency presentations.

## 1. Introduction and Clinical Significance

Upper gastrointestinal (GI) bleeding is a frequently encountered medical emergency that requires immediate attention to identify and address its underlying cause. Most cases are attributed to peptic ulcers, variceal bleeding, or esophagitis, and clinical management often revolves around fluid resuscitation, hemodynamic stabilization, and endoscopic evaluation [1,2]. However, unusual presentations necessitate a broader diagnostic approach, particularly when conventional findings do not align with the patient’s clinical condition.

This case report details a rare and unexpected diagnosis in a patient presenting with presumed upper GI bleeding, which was later identified as anaphylaxis secondary to accidental ingestion of wasps. Ingestion of stinging insects is an exceedingly rare occurrence, with systemic reactions resulting from venom absorption documented in only a handful of cases. The ingestion of live or dead wasps or bees can lead to various clinical manifestations, including localized esophageal injury, allergic responses, or, in extreme cases, systemic anaphylaxis.

Anaphylaxis is a potentially life-threatening systemic hypersensitivity reaction. It typically results from exposure to allergens such as foods, medications, or insect stings but rarely occurs due to ingestion of stinging insects [3]. The rapid absorption of venom through mucosal surfaces can precipitate profound systemic effects, including hypotension, tachycardia, and multi-organ dysfunction.

## 2. Case Presentation

A 53-year-old male patient with alcohol use disorder, with no other significant medical history, presented to our Emergency Department with recent-onset hematemesis, nausea, and dizziness. The symptoms occurred after a recent alcoholic binge. Upon physical examination, he appeared anxious, with significant hypotension (systolic blood pressure < 80 mmHg) and tachycardia (115 beats per minute). A rectal examination showed no signs of bleeding. A nasogastric suction tube was inserted, yielding aspirated red fluid with a bloody appearance. The electrocardiogram revealed sinus tachycardia, with a heart rate of 115 beats per minute, without any other significant abnormalities. The laboratory work-up revealed mild normochromic normocytic anemia (hemoglobin 11 g/dL), markedly elevated liver enzymes (AST 1400 U/L, ALT 800 U/L), and acute kidney injury (creatinine clearance of 35 mL/min/1.73 m^2^), likely attributable to either recent heavy alcohol intake or splanchnic hypoperfusion. Abdominal ultrasonography was unremarkable except for moderate hepatic steatosis without any signs of advanced liver disease or portal hypertension and a collapsed inferior vena cava, suggesting a relative hypovolemia.

A working diagnosis of presumed upper GI bleeding was established, and therapy was commenced according to the hospital’s protocol. Initial management comprised aggressive intravenous fluid resuscitation with Ringer’s solution and normal saline (2.5 mL/kg/h) and vasopressors (norepinephrine tartrate, starting with a dose of 0.1 μg/kg/min, reaching a maximum dose of 0.3 μg/kg/min) to maintain a target mean arterial pressure of 65 mmHg, along with intravenous proton pump inhibitors (pantoprazole 80 mg i.v. bolus, followed by a continuous infusion of 8 mg/h), resulting in gradual hemodynamic stabilization.

**Figure 1:**
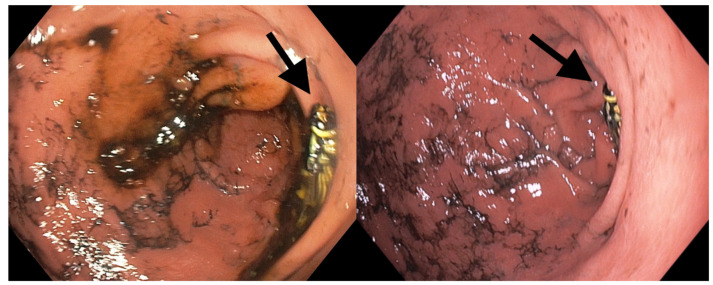
The endoscopic view of the gastric antrum, revealing the wasps, highlighted with a black arrow.

**Figure 2:**
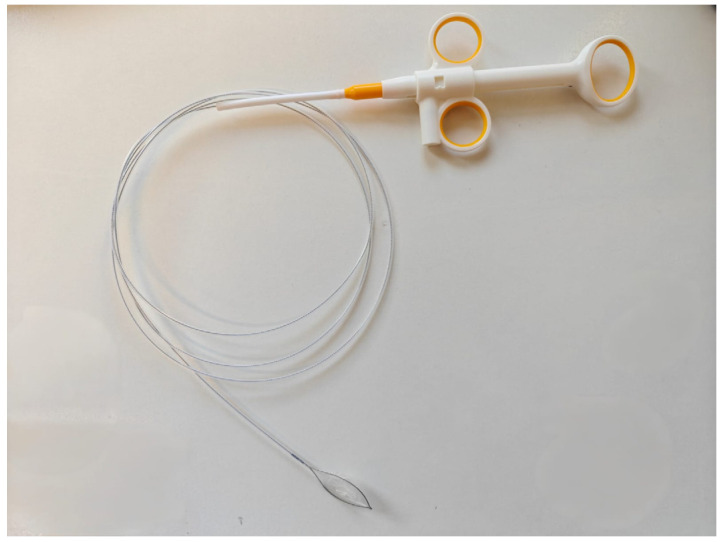
The endoscopic retrieval net is designed as a disposable snare with a tear-proof perforated extraction bag with a snare opening of 20–30 mm.

**Figure 3:**
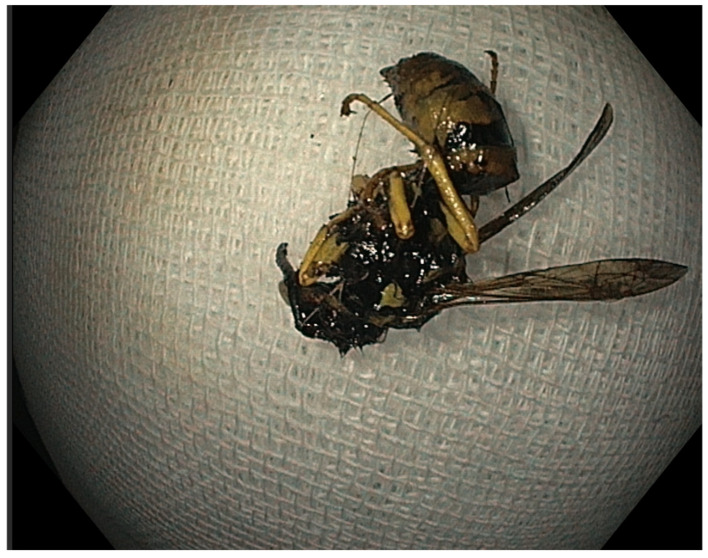
The wasp retrieved using the endoscopic retrieval net.

Given the presumed diagnosis, the patient was referred to the endoscopy department for an emergency upper GI endoscopy. The endoscopy was performed by an experienced on-call endoscopist, with a record of over ten thousand conventional endoscopic procedures and over five thousand interventional procedures (including endoscopic retrograde cholangiopancreatography, endoscopic submucosal dissection, and bariatric endoscopy) throughout his career in the largest regional tertiary care facility in the country, with over 2500 upper gastrointestinal bleeding procedures being performed annually. However, the examination did not reveal any signs of upper GI bleeding, thus contradicting the initial presumptive diagnosis. Instead, the endoscopy revealed severe reflux esophagitis (Grade C Los Angeles) with no active or stigmata of recent bleeding nor digested blood throughout the tract. Consequently, esophagitis was ruled out as a cause of presumed hematemesis and was interpreted in the context of at-risk alcohol consumption, as reported by the patient. Yet, in the antrum, we made an unexpected discovery: two intact, dead wasps (Hymenoptera) with no overt evidence of stingers embedded in the esophageal, gastric, or duodenal lining but a significant mucosal edema in the antrum (Figure 1). Using an endoscopic retrieval net (Endoaccess GmbH, Garbsen, Germany)—Figure 2, the wasps were carefully extracted (Figure 3).

Upon further investigation, the patient recounted that he had been drinking from a bottle of home-brewed alcoholic beverage (cherry brandy) shortly before the onset of his symptoms. It appeared likely that the wasps had entered the bottle, attracted by the drink’s sweetness, and were accidentally ingested. The red fluid, initially mistaken for blood, was then reinterpreted as the alcoholic beverage, and the patient’s shock symptoms were attributed to an anaphylactic reaction to wasp venom rather than hemorrhagic shock. Notably, he had no known history of allergic reactions.

Antihistaminic therapy was then promptly administered, and the patient remained under observation, showing significant clinical improvement over the next three days. His laboratory work-up steadily improved throughout the hospital stay and was interpreted in the context of anaphylaxis-related organ hypoperfusion. He was subsequently discharged in a stable condition with recommendations for allergy testing to assess venom-specific hypersensitivity and counseling for alcohol use disorder.

## 3. Discussion

Wasp, bee, and other Hymenoptera group representative stings are common occurrences and represent a common cause of allergic reactions and anaphylaxis [4]. According to recent reports, the prevalence of Hymenoptera venom allergy ranges from 0.3% to 7.5%, and is among the leading causes of presentation for acute allergic reactions [4]. The most common cause of an allergic reaction is by far a bee or wasp sting occurring during leisure or occupational exposure [5]. On the other hand, ingestion leading to severe systemic reactions, such as anaphylaxis, is extremely rare. The evidence for accidental swallowing of stinging insects is largely anectodical, as there have been only a few reported cases. Most often, ingesting stinging insects results in varying degrees of mechanical injury, such as local esophageal or gastric injury (thus behaving as a foreign body) or rare systemic anaphylaxis. After the first endoscopic depiction of a mechanical injury produced by ingested foreign bodies and the technique of endoscopic foreign body removal by Mandel et al. in 1975 [6], evidence started to emerge regarding the ingestion of Hymenoptera group representatives. In 1981, Farivar first documented a case of such accidental ingestion, ironically presenting the case of a “45-year-old executive presenting with a sudden onset of severe odynophagia followed by dysphagia, while competing with yellow jacket bees for his can of iced tea on the golf course” in a letter published in the New England Journal of Medicine [7]. In 1997, Lynch and Rothstein creatively coined the pun “gastric bee-zoar,” highlighting the unusual presentation of such cases [8], while Kesseler and Schoenemann reported an “Unusual summer-time foreign body in the esophagus” discovered during endoscopy [9]. To our knowledge, there has been a singular report of Hymenoptera ingestion leading to peptic-like symptoms (acute heartburn) and anaphylaxis to date, with a similar presentation to our case but without any suspicion of upper GI bleeding [10]. Therefore, the uniqueness of our case resides in the anaphylactic reaction to the ingestion of Hymenoptera venom, mimicking all the details of an episode of acute upper GI bleeding (vomiting of a reddish liquid resembling blood, hemodynamic instability, systemic signs of shock, and at-risk behavior for peptic ulcer disease).

The pathophysiology behind the systemic reaction in our case likely involves the rapid absorption of venom across the gastric mucosa, leading to severe anaphylaxis. Hymenoptera venom allergy is a hypersensitive immune response to insect stings from species like honeybees, wasps, or ants. The pathophysiology involves an IgE-mediated reaction wherein venom allergens activate mast cells and basophils through cross-linking of IgE bound to their FcεRI receptors. This results in the release of histamine, leukotrienes, prostaglandins, and other mediators, causing symptoms ranging from localized swelling to systemic anaphylaxis. Systemic allergic reactions occur in a subset of individuals sensitized to venom proteins, with severe cases progressing to anaphylactic shock. The venom contains components like phospholipases, hyaluronidases, and melittin, which act as allergens and cause direct tissue damage and immune activation [11].

Another point worth discussing is the factual management of such cases. In most cases of suspected upper GI bleeding complicated by hemodynamic instability, an emergency multidisciplinary team comprising an emergency physician, an intensive care specialist, a gastroenterologist with emergency endoscopy competence, and a surgeon is assembled, with the latter usually being on guard for endoscopic hemostatic failure [1]. Although, in our case, the presumptive diagnosis was unexpectedly rejected, the same team was decisive in providing the best possible outcome for our patient, albeit with the additional involvement of an allergologist throughout the patient’s hospital stay. Regarding the preparation for endoscopy, both endoscopic hemostasis and the endoscopic retrieval of foreign bodies are considered basic endoscopic techniques and can be performed by level 1 endoscopists with adequate training. According to the recommendations provided by the European Society of Gastrointestinal Endoscopy (ESGE), a core competency for upper GI endoscopy is reached after a minimum of 130 procedures, while an additional supervised 25 cases of endoscopic hemostasis provides the minimal prerequisites for managing upper GI bleeding episodes [12]. Switching to the actual diagnosis for this case, endoscopic foreign body retrieval is also considered a basic endoscopic procedure, although specific procedural cut-offs for proficiency have yet to be defined [12]. Regarding the technique per se, there is a wide array of choices for retrieving devices, ranging from endoscopic forceps to snares, retrieval nets, and protective equipment for sharp, cutting objects. Device selection depends on the shape, size, and number of objects, aiming to safely retrieve the foreign body without inflicting additional damage to the digestive tract and to prevent aspiration. According to the ESGE Guidelines for the removal of foreign bodies in the upper GI tract, the best-suited tool for cases resembling ours is the retrieval net [13]. Not least, it is essential to highlight that the advances in endoscopic techniques and armamentarium have led to a significant reduction in the need for surgery, as surgery is now limited to cases in which the foreign body cannot be safely retrieved endoscopically and poses a risk of intestinal obstruction [13]. Thus, in our case, a surgery-free, minimally invasive endoscopic approach was guaranteed to succeed, given the nature and size of the foreign bodies.

This case underscores the need for a high index of suspicion and careful history taking, especially in atypical presentations. Luckily, fluid resuscitation and the use of vasopressors and the standard of care in managing hemodynamic instability in upper GI bleeding, along with the absence of any respiratory involvement and oropharyngeal swelling, proved critical in controlling anaphylaxis in this case prior to endoscopy. The case also emphasizes the value of prompt endoscopic evaluation and strict adherence to guidelines as a systematic approach to managing hemodynamic instability in the setting of presumed GI bleeding, as the timely endoscopy proved essential in establishing the final diagnosis.

## 4. Conclusions

In conclusion, while upper GI bleeding is a common emergency, clinicians should remain vigilant for rare but serious differential diagnoses, such as insect-induced anaphylaxis. This case serves as a reminder to approach every clinical scenario with an open mind, thoroughly reviewing the patient’s history and potential environmental exposures despite strong indicators for an alternate presumptive diagnosis.

## Data Availability

The original contributions presented in this study are included in the article. Further inquiries can be directed to the corresponding author.
